# UNMET NEEDS OF VETERANS AND CAREGIVERS INCREASE WITH PREDICTED RISK OF LONG-TERM INSTITUTIONAL CARE

**DOI:** 10.1093/geroni/igae098.1092

**Published:** 2024-12-31

**Authors:** Sandra Garcia-Davis, Richard Munoz, Erin Bouldin, Jared Hansen, Bruce Kinosian, Orna Intrator, Stuti Dang

**Affiliations:** University of Miami, Miami, Florida, United States; South Florida Veteran Affairs Foundation for Research and Education, Miami, Florida, United States; University of Utah, Salt Lake City, Utah, United States; VA Salt Lake City Healthcare System, Salt Lake City, Utah, United States; Philadelphia Veterans Affairs Medical Center, Philadelphia, Pennsylvania, United States; Canandaigua VAMC and University of Rochester, Canandaigua, New York, United States; Miami VA Healthcare System, Miami, Florida, United States

## Abstract

To understand unmet needs, we contacted a random sample of Veterans receiving care at five Veterans Health Administration (VHA) sites and oversampled Veterans with a higher predicted 2-year LTIC risk. Veterans and their caregivers were asked to complete separate comprehensive surveys to assess demographic, social, psychological, functional, and physical domains; unmet needs; experience with home and community-based services; and caregiver support programs experience. Veteran and caregiver surveys were mailed in the same envelope to 20,000 Veterans. We received responses from 8,056 Veterans and 3,579 caregivers. Veteran respondents were mostly men (96.5%), over 65 years (94.9%), married (55.0%), Non-Hispanic White (75.2%), and residing in urban areas (80.7%). Veterans reported unmet needs in activities of daily living (ADLs; 15.7%), instrumental activities of daily living (IADL: 26.0%), nursing tasks (7.9%), pain management (15.1%), communicating with healthcare team (18.2%), and social support (12.1%). The most common unmet IADL needs were medication management (55.4%), housework (55.4%) and preparing meals (53.7%). Compared to Non-Hispanic Whites, Hispanics and Non-Hispanic Blacks were more likely to have unmet ADL and IADL needs. Caregivers averaged 7.3 hours of daily care (SD=5.5 hours); and helped with ADLs (42.5%), IADLs (57.2%), communicating with healthcare team (52.4%), and social needs (36.4%). Degree of need for assistance for all needs varied by predicted risk of institutionalization (PLI). Veterans in the high PLI tier reported the highest prevalence of unmet need. Both Veterans and caregivers described complex unmet needs in medical, functional, psychological, and social domains, which increased as their predicted risk for LTIC increases.

